# A Machine Learning Model Based on Global Mammographic Radiomic Features Can Predict Which Normal Mammographic Cases Radiology Trainees Find Most Difficult

**DOI:** 10.1007/s10278-024-01291-8

**Published:** 2024-10-15

**Authors:** Somphone Siviengphanom, Patrick C. Brennan, Sarah J. Lewis, Phuong Dung Trieu, Ziba Gandomkar

**Affiliations:** 1 Medical Image Optimisation and Perception Group, Discipline of Medical Imaging Science, Faculty of Medicine and Health, Sydney School of Health Sciences, Susan Wakil Health Building D18, the University of Sydney, Sydney, NSW 2006 Australia; 2 School of Health Sciences, Western Sydney University, Sydney, NSW 2751 Australia

**Keywords:** Radiomics, Mammography, Difficult normal cases, Radiology trainees, Machine learning, Gist

## Abstract

This study aims to investigate whether global mammographic radiomic features (GMRFs) can distinguish *hardest-* from *easiest-to-interpret* normal cases for radiology trainees (RTs). Data from 137 RTs were analysed, with each interpreting seven educational self-assessment test sets comprising 60 cases (40 normal and 20 cancer). The study only examined normal cases. Difficulty scores were computed based on the percentage of readers who incorrectly classified each case, leading to their classification as *hardest-* or *easiest-to-interpret* based on whether their difficulty scores fell within and above the 75th or within and below the 25th percentile, respectively (resulted in 140 cases in total used). Fifty-nine *low-density* and 81 *high-density* cases were identified. Thirty-four GMRFs were extracted for each case. A random forest machine learning model was trained to differentiate between *hardest-* and *easiest-to-interpret* normal cases and validated using leave-one-out-cross-validation approach. The model’s performance was evaluated using the area under receiver operating characteristic curve (AUC). Significant features were identified through feature importance analysis. Difference between *hardest-* and *easiest-to-interpret* cases among 34 GMRFs and in difficulty level between *low-* and *high-density* cases was tested using Kruskal–Wallis. The model achieved AUC = 0.75 with *cluster prominence* and *range* emerging as the most useful features. Fifteen GMRFs differed significantly (*p* < 0.05) between *hardest-* and *easiest-to-interpret* cases. Difficulty level among *low-* vs *high-density* cases did not differ significantly (*p* = 0.12). GMRFs can predict *hardest-to-interpret* normal cases for RTs, underscoring the importance of GMRFs in identifying the most difficult normal cases for RTs and facilitating customised training programmes tailored to trainees’ learning needs.

## Introduction

Female breast cancer (BC) stands as one of the leading causes of cancer-related mortality worldwide [1]. Mammographic screening programmes, aimed at early detection of BC, have been shown to significantly reduce BC mortality rate by up to 40% [2]. However, interpreting mammograms is a challenging task susceptible to errors, and relying primarily on technological advancements is insufficient to mitigate or address these challenges [3]. Previous studies have reported that correctly interpretating normal cancer-free mammograms can also be highly difficult for readers with inadequate mammographic reading experience such as radiology trainees (RTs) [4, 5]. The capability of readers including RTs to accurately interpret screening mammograms plays a pivotal role in the efficacy of breast screening programmes. Despite efforts to propose criteria for screen reader certification aimed at reducing diagnostic variability among readers, none of the current guidelines for these criteria serve as strong predictors of reader performance [6, 7]. As a result, wide inter-reader variability persists, highlighting the critical need to prioritise education and training for readers, especially for less experienced readers like RTs, in this domain [6–9]. Given that normal cases comprise over 99% of all cases in breast screening programmes [10, 11], along with the fact that false-positive screening results cause huge healthcare costs of nearly US$3 billion yearly (in the USA as an example) and psychological distress to patients due to unnecessary recalls involving invasive procedures [12], it is imperative that education strategies are prioritised to give extra attention to normal cases.

Studies from medical image perception and basic vision science demonstrate that exposing RTs to large mammographic normal cases via test set training programmes, often containing nearly 70% of normal cases in a test set, can enhance their expertise development in interpreting normal cases [13–15]. Still, the present test set curation process using experts’ or individual’s perception of case difficulty via the breast imaging and reporting data system (BI-RADS) breast density and/or benign features are unreliable predictors of normal cases’ difficulty among RTs [11, 16–18]. Therefore, there is a strong demand for a more systematic approach of predicting RTs’ difficult normal cases so that customised mammographic training test sets for RTs can be facilitated while their false positive errors can be better monitored and improved.

The process of interpreting medical images necessitates the development of capabilities for globally processing images. Earlier studies showed that individuals with less experience such as RTs often lack the ability to swiftly process the global clues indicative of abnormalities [19]. To investigate this further, we designed an experiment protocol to gather the initial impressions of radiologists regarding the abnormality of a case, known as the gist signal [19–21]. This signal not only indicates a current cancer case [22] but also serves as a predictor of future BC [21]. Moreover, recent research in radiomics has shown that quantitative global mammographic radiomic (or computer extract image) features align closely with the holistic perceptual information gleaned from radiologists’ rapid first impressions about the presence of an image abnormality or global gist signal [20, 23]. This signal has proven valuable in guiding visual searches and aiding in making diagnostic decisions when interpreting challenging mammographic cases [11, 22, 24].

Radiomic features capture various aspects of the mammogram, including texture, intensity, and spatial relationships, providing comprehensive information about the underlying tissue characteristics [25, 26]. Radiomic features have been the subject of systematic investigation in previous works, where local radiomic features were utilised to identify missed mammographic masses and false positive location errors of RTs, achieving area under receiver operating characteristic curves (AUCs) from 0.59 to 0.61 and accuracies from 12 to 40%, respectively [4, 5]. More recent studies [11, 16] have revealed that using global mammographic radiomic features (GMRFs), the most difficult/easiest normal cases for readers in general and experienced radiologists can be accurately predicted (AUCs of up to 0.73). However, to date, there has been no study exploring whether GMRFs can specifically predict *hardest-* from *easiest-to-interpret* normal cases for RTs. Considering the fact that RTs are still developing their holistic processing capabilities compared to experienced radiologists [13, 21, 22, 24], this paper explores whether GMRFs can serve as reliable predictors specifically for *hardest-to-interpret* normal cases for RTs. It also seeks to recognise if there are any specific significant GMRFs that denote the *hardest-to-interpret* normal cases for RTs. Knowing these could be valuable not only in the training programs’ test set curation process of most difficult normal cases for RTs but also facilitating their expertise development in recognising normal image features that could be most difficult for them often resulting in false positive errors. This is so that their specific skills in perceiving the global signature of normality can be well developed since RTs are generally not as good at recognising the normal features as compared to experienced radiologists [13, 24, 27].

Furthermore, since the current approach to curating test sets relies on experts’ opinions regarding case difficulty, primarily using breast density as a surrogate metric [11, 16–18], we also examine if a significant difference exists in difficulty level between *low-density* (BI-RADS category A = fatty and B = fibroglandular) and *high-density* (category C = heterogeneous and D = extremely dense) normal cases.

## Materials and Methods

### Readers

Ethical approvals of this research were acquired from the University of Sydney’s Human Research Ethics Committee [protocol no. 2019/013 and 2017/028], which included obtained written informed consent from each reader participating in the study.

A total of 137 Australian and New Zealand RTs who participated in the BREAST (Breastscreen REader Assessment STrategy) programme [28] from 04 September 2014 to 23 February 2021 were included in the study. The mean age of the readers was 33 based on the age details provided by 116 RTs, while 21 did not provide their age details. In total, 19% of the readers engaged in the programme through a radiology/BC conference/workshop in a simulated reading room, while 81% completed the programme online through the BREAST platform (https://breast-australia.sydney.edu.au/) at their usual clinical workplace. Reading conditions in both settings were equivalent with cases being presented on two 5-megapixel medical standard monitors including ambient light levels range of 20–40 lx [29]. RTs generally had less than 1 year experience reading mammograms (59%), read less than 20 mammographic cases per week (79%), and spent less than 4 hour per week reading mammograms (96%). Also, 16 readers (12%) completed a fellowship in breast imaging that lasted for 3 to 6 months and had any experience reading images for a national breast screening programme (Table 1).

**Table 1:** Characteristics of readers (*n* = 137)

Mean years of age (derived from 116 radiology trainees’ age details while 21 radiology trainees did not provide their age details)	33	
	***N***	**%**
*Country*		
Australia	135	99
New Zealand	2	1
*Location at the time of engaging with the BREAST programme*		
At a workshop or conference	26	19
At a clinic	111	81
*# of years reading mammograms*		
Less than 1	81	59
1 to 5	53	39
6 to 12	3	2
*# of mammographic cases reading per week*		
Less than 20	108	79
20 to 200	28	20
More than 200	1	1
*# of hours per week spent reading mammograms*		
Less than 4	131	96
4 to 30	5	4
More than 30	1	1
*# of readers completed a fellowship lasted for 3 to 6 months*		
Completed fellowship	16	12
Did not complete fellowship	119	87
Not specified	2	1
*# of readers read for breast screening programme*		
Yes	16	12
No	121	88

### Mammographic Normal Cases

This retrospective study includes data from readers that completed seven BREAST test sets, each comprising 60 de-identified full-field digital mammographic (FFDM) cases, with 40 cases being cancer-free and 20 cases indicating cancer. For the purpose of this investigation, only normal cancer-free cases were included. Thus, a total of 280 normal FFDM cases (comprised of four images per case: two bilateral craniocaudal/CC and two mediolateral oblique/MLO views) acquired from screening asymptomatic women aged between 40 and 75 (mean and median = 57, standard deviation = 7, and range = 33 based on the age details of 130 women/cases, while ages of 10 women were not available due to anonymisation process) were utilised. These cases were collected from various mammography machines, including Fujifilm (Fujifilm Corporation, Minato City, Tokyo, Japan), GE (GE Healthcare, Chicago, IL, USA), Hologic (Hologic, Inc., Marlborough, MA, USA), Philips (Philips Healthcare, Amsterdam, the Netherlands), Sectra (Sectra, Linköping, Sweden), and Siemens (Munich, Germany). Each normal case’s ground truth underwent confirmation of cancer-free status by at least two independent expert radiologists, each possessed over 20 years of experience, with validation through subsequent negative screen outcomes.

### Categorising Hardest- vs Easiest-to-Interpret Normal Cases 

To categorise normal cases as *hardest-* vs *easiest-to-interpret*, we initially calculated difficulty scores for each of the 280 normal cases using the Royal Australian and New Zealand College of Radiologists (RANZCR) scoring system [28] provided by the 137 RTs. A RANZCR score of 1 represents a normal case, while a score of 2 indicates that RTs considered the case as benign. Scores of 3, 4, or 5 signify an equivocal or malignant finding observed by the readers with higher numbers indicating a greater confidence of disease presence. Difficulty scores were computed by dividing the number of RTs who misclassified a normal case as cancer (provided a rating of 3, 4, or 5) by the total number RTs who read the test set. Subsequently, the 280 normal cases were then classified as *hardest-* and *easiest-to-interpret* cases based on the 75th and 25th percentiles of the cases containing the highest and lowest difficulty scores, correspondingly. This resulted in 70 *hardest-* and 70 *easiest-to-interpret* normal cases, totalling of 140 normal cases/560 DICOM images combined. To ensure a clear distinction between the most and least difficult normal cases in order to enable optimal feature learning for our machine learning model, only images in the upper and lower quartiles were used for further analysis. Normal cases in these two categories also resulted in a total of 59 *low-density* (35 *hardest-* and 24 *easiest-to-interpret*) and 81 *high-density* normal cases (35 *hardest-* and 46 *easiest-to-interpret*).

### Global Radiomic Feature/Score Per Case

**Fig. 1:**
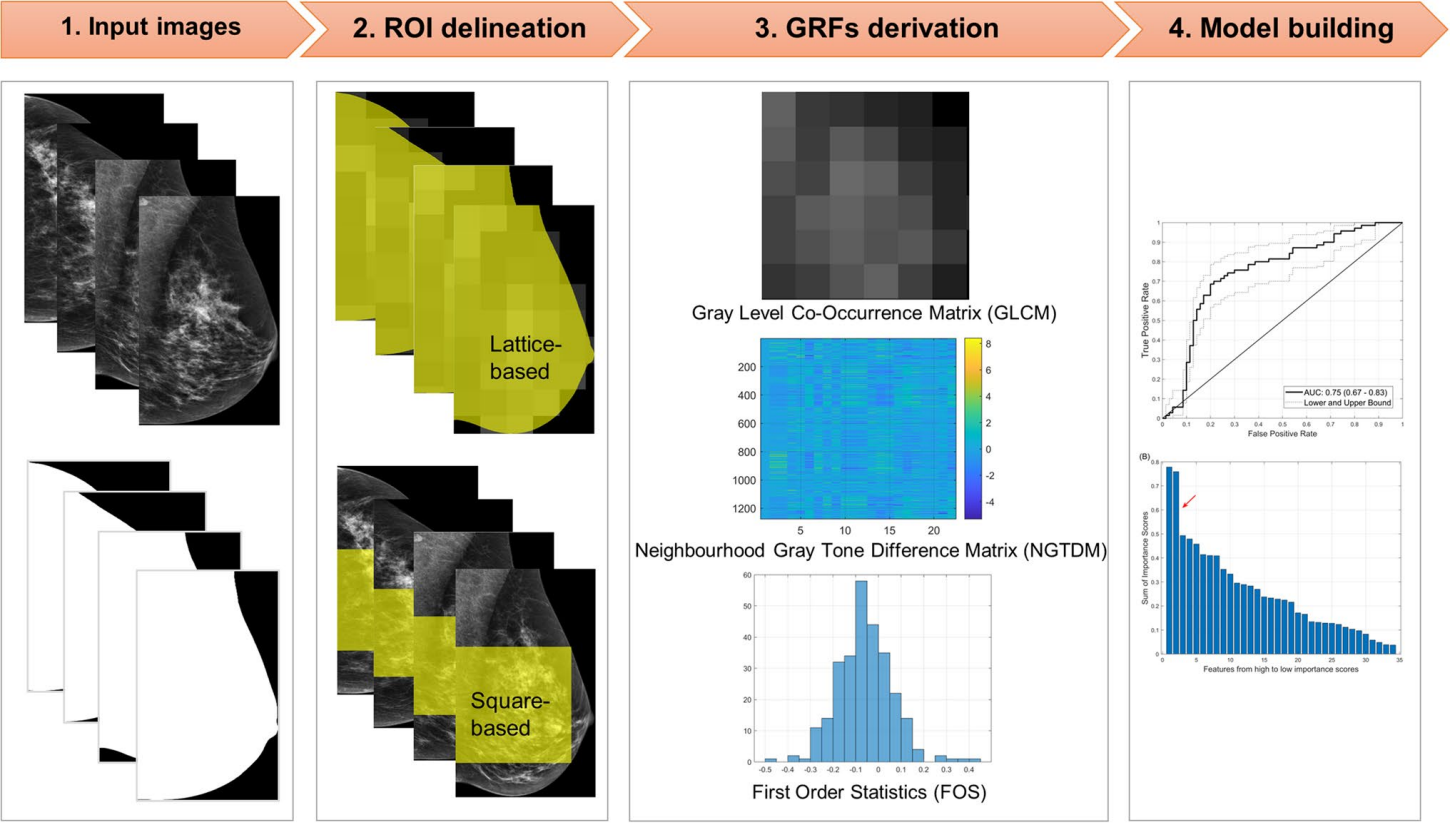
Radiomics workflow. **1.** Input mammographic images and masks were acquired. **2.** Region of interest (yellow colour region) was delineated using lattice- and squared-based approaches. **3.** A set of 34 global mammographic radiomic features (GMRFs)/scores per image were extracted from the yellow region of interest, then standardised to have image intensity mean equal to zero and standard deviation equal to one. The extracted GMRFs of four images belonging to each of the 140 cases were then averaged to obtain one GMRF per case. **4.** Finally, using the averaged GMRFs, a random forest machine learning model for differentiating radiology trainees’ *hardest-* from *easiest-to-interpret* normal cases was constructed and assessed

To obtain a global radiomic feature/score for each of the 140 normal cases, a total of 34 quantitative GMRFs of 560 images belonging to 140 cases extracted from the previous study [11] were used. Details about the GMRFs extraction method can be found in the previous study [11]. Briefly, from the 560 DICOM images, we first generated 560 binary masks using a standardised gray level threshold value set at 100 (manual adjustments on the value were also performed where needed) to extract the required breast region from its background while eliminating unwanted artefacts and labels. After that, we converted the DICOM images and masks to a TIFF file format, flipped all the right CC and MLO images to the left to have a consistent left-side chest wall on all images, and cropped the TIFF images and masks based on the maximum size of breast region. These cropped TIFF images and masks were used as input for the radiomic analysis (Fig. 1). We then outlined the region of interest on the images using the lattice-based/ROI (multiple regions of interests covering the entire breast image) [30] and squared-based/SQ (largest square rectangular box inscribed within breast) [31] approaches. The 34 handcrafted GMRFs/scores per image were afterward extracted based on the region of interest delineated using our in-house MATLAB platforms and then normalised to calibrate image intensity mean to zero and standard deviation to one using a common z-score normalisation method [32]. Features using the ROI approach [30] were analysed using MATLAB distinct block processing technique (block size 214 × 214 pixels) and summarised using the standard deviation method. The extracted GMRFs of 4 images (i.e. 2 CC and 2 MLO images) belonging to a case were then combined and averaged to obtain one global radiomic feature/score per case.

The 34 GMRFs consisted of 30 Gray level co-occurrence matrix/GLCM-based Haralick texture features [33], 2 neighbourhood gray tone difference matrix/NGTDM-based texture features [34], and 2 first-order statistics/FOS-based features [25] (Table 2 and Fig. 1). These features were chosen because of their usefulness in describing mammographic appearances in measuring the contrast values of spatial inter-relationships between neighbouring pixels (GLCM and NGTDM) and the distribution of single pixel intensity value within the image region of interest (FOS) [25, 33].

**Table 2:**
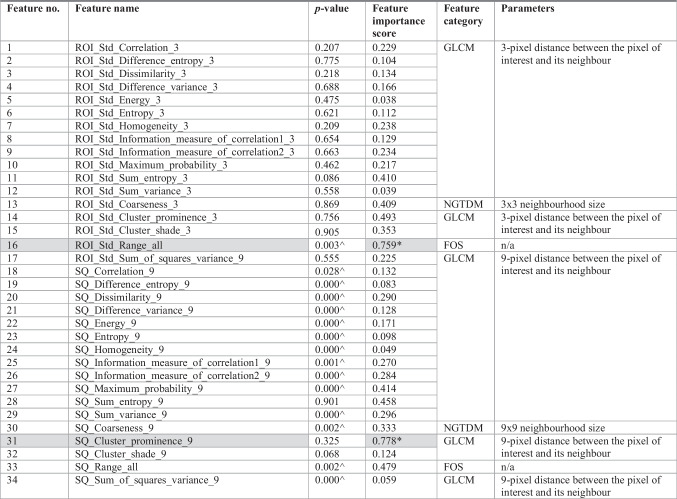
Extracted global mammographic radiomic features (*n* = 34)

^Features (*n* = 15) showing statistically significant difference (*p*-value < 0.05) between *hardest-* and *easiest-to-interpret* normal cases of radiology trainees

^*^ and highlighted in grey = important features (*n* = 2) from the machine learning classifier. The higher importance score means the more useful the features were for the classifier

Abbreviations: *FOS* first order statistics, *GLCM* gray-level co-occurrence matrix, *NGTDM* neighbourhood gray-tone difference matrix, *n/a* not applicable, *ROI* multiple regions of interests defined by the lattice-based approach covering the entire breast image, *SQ* largest square rectangular box inscribed within breast, *Std* standard deviation

### Model Building

For the task of differentiating between normal cases that are *hardest-* and those of *easiest-to-interpret* for RTs, a random forest machine learning model was built using the averaged 34 GMRFs feeding through MATLAB ensemble of decision trees boosted with the adaptive logistic regression method (i.e. LogitBoost). The random forest with boosting approach was chosen due to its ability to produce an explainable model with automatic estimation of feature importance and its built-in feature selection approach which could minimise feature overfitting and potential selection bias problems [35]. Important GMRFs were recognised based on the analysis of feature importance scores using MATLAB’s predictor importance algorithm, which also helps in identifying and mitigating redundant and biased features. Feature importance scores indicate how significant each feature was in the dataset when building the predictive model with larger value means larger effect the features had on the model in predicting *hardest-* from *easiest-to-interpret* normal cases of RTs (Fig. 1).

### Statistical Analysis and Validation

In order to examine the performance of the model on our data and to maximise the use of the available data for both training and validation, we trained and validated the model using the resampling leave-one-out-cross-validation (LOOCV) approach (an unbiased, dependable, and accurate validation method for assessing a machine learning model’ generalisation performance) [36]. Each time when training the model, all cases were used, except one case which was left out and used once as a test set to validate the model’s predictive performance. We repeated this process 140 times (as per the total number of the normal cases we had) until each case was left out and used once as a test set. The model’s performance was then evaluated based on how accurately it predicted the left-out/unseen case in each repetition, providing an effectively estimation of how well the model generalises to the unseen cases. The overall performance of the model for discriminating *hardest-* from *easiest-to-interpret* normal cases of RTs was assessed using the AUC.

A Kruskal–Wallis test was employed to investigate if the 34 GMRFs differed between *hardest-* and *easiest-to-interpret* normal cases of RTs, and if difficulty level differed among *low-* vs *high-density* normal cases of RTs. A *p*-value of less than 0.05 was considered statistically significant.

Moreover, a scree test of exploratory factor analysis [37] was used to determine the usefulness of the GMRFs based on the sum of their importance scores from the model.

Radiomics and statistical analysis were performed using MATLAB R2022a (MathWorks, Natick, MA, USA).

## Results

### Global Mammographic Radiomic Features Predicting Difficult Normal Cases of Radiology Trainees

**Fig. 2:**
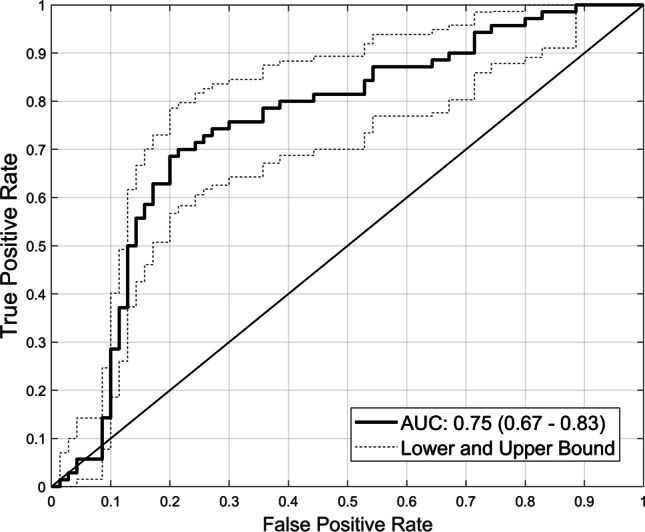
Model performance. For discriminating *hardest-* from *easiest-to-interpret* normal cases of radiology trainees, AUC = 0.75 (95% confidence interval, 0.67– 0.83) was achieved by the random forest machine learning model

The overall performance of the machine learning classification model for differentiating *hardest-* from *easiest-to-interpret* normal cases of RTs using GMRFs is shown in Fig. 2, which indicates an AUC of 0.75 (95% confidence interval, 0.67–0.83).

### Significant Global Mammographic Radiomic Features and Breast Density of Difficult Normal Cases

Table 2 shows that 15 out of the total 34 GMRFs had a statistically significant difference between *hardest-* vs *easiest-to-interpret* normal cases of RTs with *p* < 0.05.

**Fig. 3:**
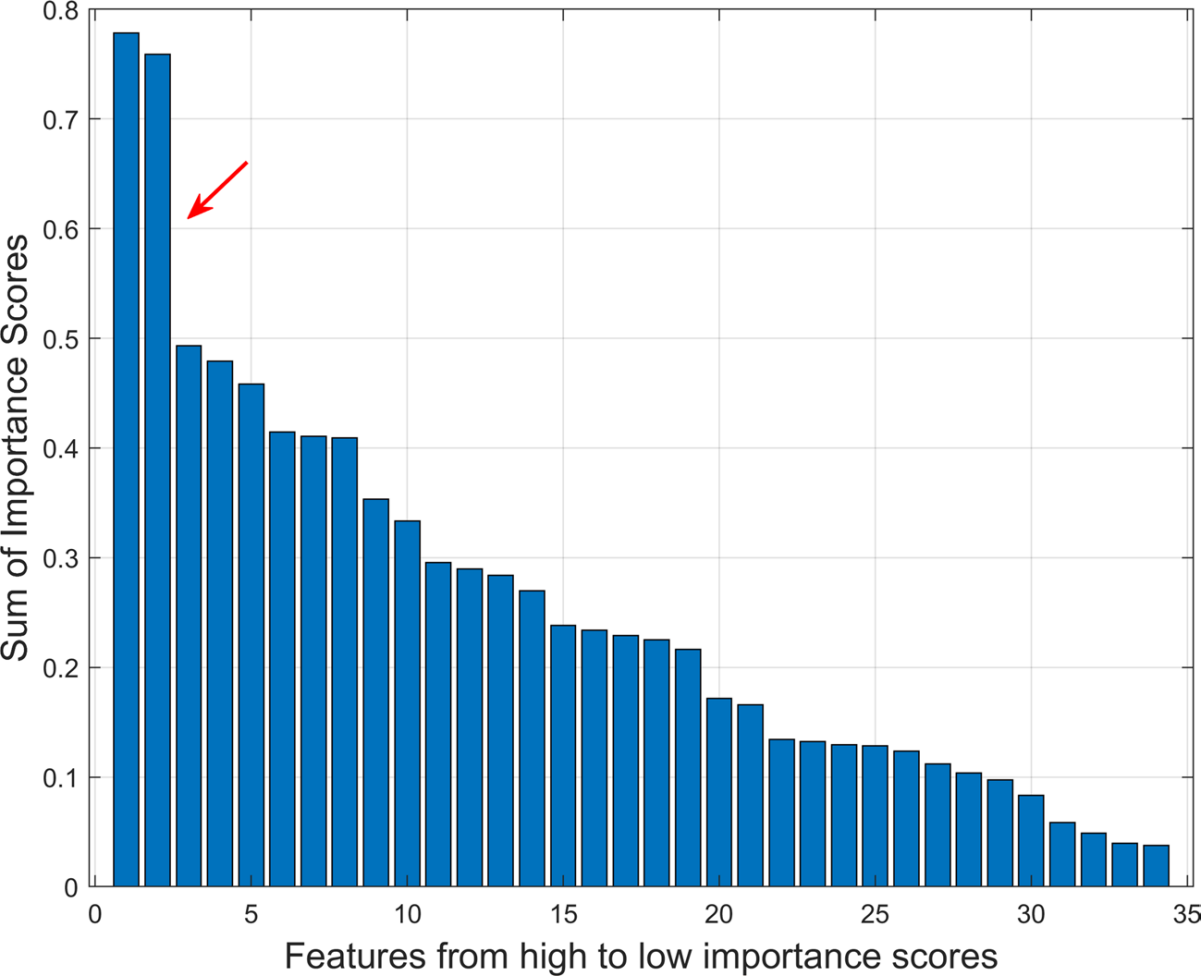
Scree plot showing significant global mammographic radiomic features. From 34 features, two important features were identified, indicating in the scree plot with the steep slope occurred after the second highest features (as shown by the red arrow). Those important features, ranked from the highest to the lowest important scores, were: (1) *SQ_Cluster_prominence_9* (feature# 31) of GLCM, and (2) *ROI_Std_Range_all* (feature# 16) of FOS. FOS first order statistics, GLCM gray-level co-occurrence matrix, ROI multiple regions of interests defined by the lattice-based approach covering the entire breast image, SQ largest square rectangular box inscribed within breast

Table 2 and Fig. 3 show the significant GMRFs based on their importance scores derived from the predictive machine learning model. Out of the 34 features, when comparing each feature’s total importance scores ranking from the highest to the lowest, two important features were recognised. These features were *SQ_Cluster_prominence_9* (feature# 31) of GLCM, and *ROI_Std_Range_all* (feature# 16) of FOS. *Cluster prominence* measures the skewness and asymmetry of the GLCM [33] and was calculated using the squared-based approach [31]. *Range* describes the difference between maximum and minimum of image gray level values [25] and was calculated using the lattice-based method [30].

**Fig. 4:**
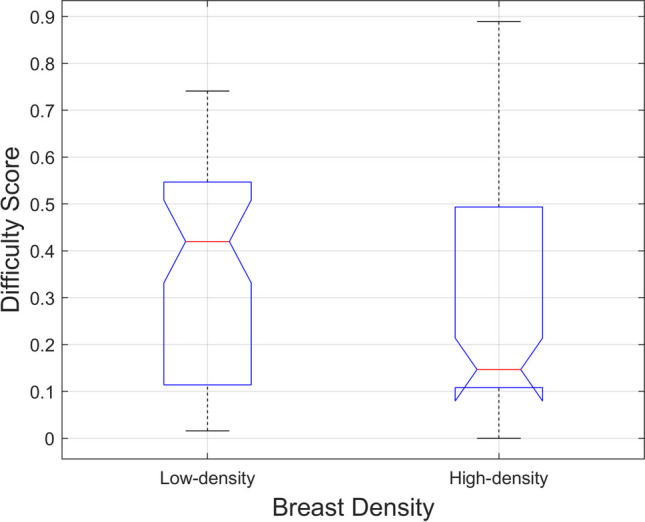
Breast density comparison. Non statistically significant difference (*p* = 0.12) in the difficulty level was found between *low-density* and *high-density* normal cases of radiology trainees

Figure 4 reveals that the difficulty of fatty or *low-density* normal cases does not significantly differ from that of dense or *high-density* cases (*p*-value = 0.12), with *low-density* cases appearing to be more challenging for RTs, albeit non-significant.

## Discussion

In this proof-of-concept study, we explored (1) if using a set of global handcrafted radiomic or computer-extracted image features derived from mammograms, *hardest-* vs *easiest-to-interpret* normal cancer-free cases of RTs can be classified, and (2) whether any of these features are useful in this classification task.

Previous works [4, 5] recognised the usefulness of local radiomic features in identifying missed mammographic masses and false positive location errors of RTs, obtaining AUCs of up to 0.61 and accuracies of up to 40%. Other later studies [11, 16] indicated the value of GMRFs in predicting most *difficult-to-interpret* normal cases of general and experienced radiologists (over 20 years of experience reading mammograms), attaining AUCs of up to 0.73. Building on these recent research outputs [11, 16], we explored the feasibility of using GMRFs in describing RTs’ *hardest-* vs *easiest-to-interpret* normal cases based on the hypothesis that the image characteristics of these two categories’ cases are diverse. In addition, the results from our machine learning classifier revealed similar findings in that GMRFs (describing the whole image and largest square rectangular inscribed within breast) had the ability to distinguish between *hardest-* from *easiest-to-interpret* normal cases of RTs, achieving an AUC of 0.75 (95% CI, 0.67–0.83) (Fig. 2). This finding affirms the importance of GMRFs in predicting *hardest-to-interpret* normal cases for less-experience readers like RTs (Table 1).

Mammographic breast density using BI-RADS category are often employed as a surrogate measure in assessing difficult normal cases for RTs, where cases with higher density are regularly considered to be more difficult to interpret [17, 38]. However, studies have shown that breast density category details alone may not be completely reliable for predicting difficult/easy normal cases of radiologists including RTs [11, 39]. In line with these findings, the results from our dataset confirmed that BI-RADS density categories were not strongly correlated with difficulty scores (Fig. 4). Our GMRFs (e.g. *SQ_Cluster_prominence_9* and *ROI_Std_Range_all*) relating to *hardest-to-interpret* normal cases of RTs therefore may be used to provide supplementary clinical image information (in addition to BI-RADS breast density class) about most difficult features that RTs are likely to commit a false positive error. Also, since RTs find recognising normal image features challenging [13, 14], our findings could therefore aid their screening mammographic interpretation expertise development in recognising hardest and easiest breast features of normal cases which could then facilitate automatic hardest and easiest normal cases test set curation for customised education/training programmes. Using the proposed model, we can create customised test sets tailored to the specific learning needs of trainees at different stages of their careers. For example, early-stage trainees could be presented with easier cases to build foundational skills, while more advanced trainees could be challenged with harder cases that reflect real-world complexities. This customised test set curation would allow for targeted education and training programmes that align with the trainees’ current skill levels, helping them to progress more effectively through their learning journey.

Interestingly, when comparing two groups based on a single GMRF, 15 out of 34 features showed a statistically significant difference between *hardest-* and *easiest-to-interpret* normal cases of RTs (*p* < 0.05). However, when examining the 34 features together through our machine learning classifier, only two features (*cluster prominence*, i.e. *SQ_Cluster_prominence_9*, calculated using the squared-based approach [31] and *range*, i.e. *ROI_Std_Range_all*, calculated using the lattice-based method [30]) were found to be most useful in describing RTs’ *hardest-* from *easiest-to-interpret* normal cases (Table 2 and Fig. 3). *Range*, in particular, has also been discovered to be a valuable feature in previous studies [11, 20] highlighting the significance of *range* in various classification tasks among different groups of readers (e.g. categorising *hardest-* from *easiest-to-interpret* normal cases of readers in general as well as RTs). While *cluster prominence* was not found to be an important feature in the previous work [11, 20], in this dataset, we observed that more difficult cases, which resulted in false positive errors more frequently, exhibited higher *cluster prominence* which is a measure of the skewness and asymmetry of the GLCM. This suggests that *cluster prominence* may be an important marker of highly challenging cases for RTs. A higher value of *cluster prominence* implies more asymmetry about the mean in the GLCM, while a lower value indicates a peak near the mean value and less variation about the mean. Intuitively, *cluster prominence* refers to the extent to which clusters of pixels within an image stand out from their surroundings. In other words, it measures the distinctiveness of clusters within the image with a higher *cluster prominence* value indicating that clusters are more salient or prominent within the image. In the context of our study, normal cases with higher *cluster prominence* value pose greater difficulty for trainees. Trainees may struggle with these cases because the heightened prominence of clusters within the image makes it more challenging to distinguish between normal tissue and potential abnormalities, resulting in false positive annotations. Although the features may be abstract in nature and not quantifiable by humans, they could be quantitatively measured using our radiomics approach and used as educational tools to teach and/or alert RTs that the higher the value of *cluster prominence*, the harder the case could be for RTs, increasing the chance of them making a false positive error. Furthermore, creating an alert system based on these features and educating RTs about the correlations between higher value of *cluster prominence* and harder cases can improve their ability to recognise difficult normal cases and enhance decision-making in clinical practice. Nonetheless, more studies are needed to further examine the effectiveness and reliability of these features.

This study also has a few limitations that should be recognised. It was a proof-of-concept study examining the efficacy of 34 handcrafted GMRFs [11] in identifying the *hardest-* from *easiest-to-interpret* confirmed cancer-free normal cases of 137 RTs (mostly with less than 1 year of experience reading mammograms) specifically. Only normal cases were included in the study which contained only those that had difficulty scores of greater than or equal to the 75th percentile (*hardest-to-interpret*) and less than or equal to the 25th percentile (*easiest-to-interpret*). LOOCV approach [36] was used to train and validate the model in order to maximise the use of the available data. The GMRFs were extracted from the region of interest delineated using the lattice-based [30] and squared-based [31] approaches. Future research should explore other approaches of identifying region of interest (e.g. retroareolar region), validating the model (e.g. holdout method [36]), employing other difficult normal cases with difficulty scores falling outside the included range (e.g. scores that are less than the 75th percentile and greater than 25th percentile), and radiomic analysis involving local and/or deep learning features [26, 40].

## Conclusions

In conclusion, in addition to previous research [11, 20], our work highlights the importance of GMRFs for classifying *hardest-* from *easiest-to-interpret* normal cases of RTs with two important features (c*luster prominence* and *range*) being identified. Our findings could be useful for developing RTs’ interpretation expertise in identifying the most difficult image features of normal breast cases as well as enabling automatic and tailored test set curation of the most difficult cases for RTs’ education/training programmes.

## Data Availability

The data that support the findings of this study are available from BREAST, but restrictions apply to their availability, as they were used under license for the current study and are not publicly available. However, data may be available from the authors upon reasonable request and with permission from BREAST.

## Abbreviations

AUC: Area under receiver operating characteristic curves
BC: Breast cancer
BI-RADS: Breast imaging and reporting data system
BREAST: Breastscreen REader Assessment STrategy
CC: Craniocaudal
FOS: First-order statistics
GLCM: Gray level co-occurrence matrix
GMRFs: Global mammographic radiomic features
MLO: Mediolateral oblique
NGTDM: Neighbourhood gray-tone difference matrix
RANZCR: Royal Australian and New Zealand College of Radiologists
ROI: Multiple regions of interests defined by the lattice-based approach covering the entire breast image
RTs: Radiology trainees
SQ: Largest square rectangular box inscribed within breast
